# Computational models for *in-vitro *anti-tubercular activity of molecules based on high-throughput chemical biology screening datasets

**DOI:** 10.1186/1471-2210-12-1

**Published:** 2012-03-31

**Authors:** Vinita Periwal, Shireesha Kishtapuram, Vinod Scaria

**Affiliations:** 1 GN Ramachandran Knowledge Center for Genome Informatics, Institute of Genomics and Integrative Biology (CSIR), New Delhi 110007, India; 2 Open Source Drug Discovery Consortium, Council of Scientific and Industrial Research (CSIR, India), New Delhi, India

## Abstract

**Background:**

The emergence of Multi-drug resistant tuberculosis in pandemic proportions throughout the world and the paucity of novel therapeutics for tuberculosis have re-iterated the need to accelerate the discovery of novel molecules with anti-tubercular activity. Though high-throughput screens for anti-tubercular activity are available, they are expensive, tedious and time-consuming to be performed on large scales. Thus, there remains an unmet need to prioritize the molecules that are taken up for biological screens to save on cost and time. Computational methods including Machine Learning have been widely employed to build classifiers for high-throughput virtual screens to prioritize molecules for further analysis. The availability of datasets based on high-throughput biological screens or assays in public domain makes computational methods a plausible proposition for building predictive models. In addition, this approach would save significantly on the cost, effort and time required to run high throughput screens.

**Results:**

We show that by using four supervised state-of-the-art classifiers (SMO, Random Forest, Naive Bayes and J48) we are able to generate *in-silico *predictive models on an extremely imbalanced (minority class ratio: 0.6%) large dataset of anti-tubercular molecules with reasonable AROC (0.6-0.75) and BCR (60-66%) values. Moreover, these models are able to provide 3-4 fold enrichment over random selection.

**Conclusions:**

In the present study, we have used the data from *in-vitro *screens for anti-tubercular activity from a high-throughput screen available in public domain to build highly accurate classifiers based on molecular descriptors of the molecules. We show that Machine Learning tools can be used to build highly effective predictive models for virtual high-throughput screens to prioritize molecules from large molecular libraries.

## Background

Tuberculosis (TB) caused by *Mycobacterium tuberculosis*, is one of the major causes of morbidity and mortality in the developing world. Tuberculosis manifests in many clinical forms from an active disease state to clinical latency that can extend for decades. It has been reported that there have been 9.4 million new cases of TB with an estimated global mortality of 1.7 million in the year 2009 [1]. Recent reports suggest that a high proportion, averaging about 85% cases have been accounted to occur in Asia and Africa with India and China alone accounting to 50% of the total burden of disease [2]. Evidences also point to an increasing incidence of drug-resistant TB over the past decade [3]. In addition, its catastrophic synergy with Acquired Immune Deficiency Syndrome (AIDS) have made it a major health concern worldwide [4]. The present drugs used in the first line therapy of tuberculosis has been discovered at least half a century ago, and the unabated global rise of tuberculosis calls for the development of novel tools and methods for fast and efficient identification of novel molecules with anti-tubercular activities.

With the lack of comprehensive systems level understanding of the causative organism and its intricate biological pathways and control mechanisms, it has been suggested that whole cell phenotypic screens offer a better proposition in comparison with single gene based biological screens [5]. The availability of methods for high-throughput screening has significantly contributed for many such datasets being made available in public domain. With the very low hit rate for such high-throughput biological screens, it has become inevitable to prioritize molecules to be taken up for biological screens. It has been suggested out that virtual screening of large compound libraries using computational methods like machine learning techniques could be efficiently employed as a complementary approach to phenotypic screens in drug discovery [6-12]. The availability of small molecule bio-assay datasets in public domain provide a valuable means to build predictive computational models that can be potentially used to prioritize molecules for biological assays from large digital databases [13,14].

We have previously [15] used machine learning approaches to classify inhibitors identified from bioassay screens of *Mycobacterium tuberculosis *in Middlebrook 7H12 broth [16,17]. The 7H12 media (ADC (albumin, dextrose and catalase) enriched Middlebrook 7H9 broth) allows rapid recovery of mycobacteria from clinical specimens, sputum, and respiratory secretions. While the ADC enrichment provides more sensitivity to the microbial culture, albumin acts as a protective agent by binding free fatty acids, which may be toxic to mycobacterium species and catalase destroys toxic peroxides that may be present in the medium and dextrose acts as an energy source. In our present study we make use of a confirmatory screen that identifies novel anti-tubercular inhibitors of *Mycobacterium tuberculosis *in 7H9 broth supplemented with glycerol and tween 80 for improved growth; the media is principally used for growth of axenic cultures of mycobacteria. The library of compounds used in current bioassay excluded known inhibitors from previously pursued compounds and their analogs, on which our earlier study was based.

Although classification methods using machine learning approach are valuable tools in rapid virtual screening of compound libraries [12,18], they have been seldom used in TB drug discovery programmes [19-22]. Our present work marks an effort in this direction to make predictive models for prioritization and/or discovery of novel active molecules that can be taken up further in the drug discovery pipeline for tuberculosis.

## Results and discussion

The dataset (AID449762) used in this study is a confirmatory bioassay screen to identify novel compounds that inhibit *Mycobacterium tuberculosis *in 7H9 media. The dataset consists of 3,27,561 tested compounds with 1937 actives, 3,12,901 inactives and rest are inconclusive compounds. Inconclusive compounds were not considered in this study to avoid uncertainty in the predictive ability of the generated models. A total of 179 descriptors were calculated (this information can be found at the following link http://genome.igib.res.in/Mtb_7H9/Additional_file1.zip) and data processing was done as described in the "Methods" section. After removing un-informative bit string descriptors (i.e. the ones containing only 0's or 1's throughout the dataset), only 154 descriptors remained and were used for further classification and analysis. The list of descriptors removed after data processing is provided in Additional file 1: Table S1. The processed file was then split into training and test sets (this information can be found at the following link http://genome.igib.res.in/Mtb_7H9/Additional_file3.zip). The training set file was converted to ARFF format and loaded in Weka. As the file size was very large, Weka was started with a heap size of 8 GB to handle Out-Of-Memory exception.

Initial classification experiments were done with standard base classifiers only. All the models obtained with the base classifiers had an FP rate well below our threshold limit i.e. 20% however the resulting high accuracies were not a good representation of our dataset because it is highly imbalanced, so cost sensitivity was introduced using cost matrix to produce a more reliable predictive ability of the classifier in use. Misclassification cost for False Negatives was raised incrementally so as to stay in the upper limit of False Positives. Thus a number of models were trained based on differential cost settings. The FN cost that resulted in the best predictive models for each of the individual classifiers is depicted in Table 1.

**Table 1:** Misclassification cost used for false negatives with each classifier

Classifier	Cost
**SMO**	110
**Random Forest**	14000
**Naïve Bayes**	35
**J48**	350

**Figure 1:**
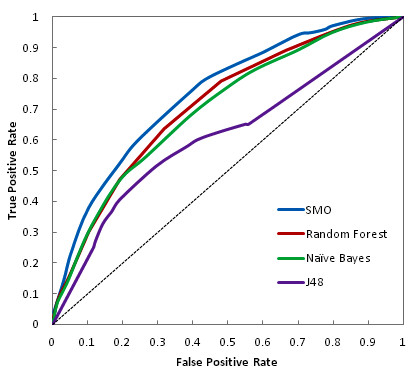
**Receiver operating characteristic (ROC) curve plot of all the models**. A plot of ROC curve for all the classifiers. Among all classifiers, SMO achieved the maximum value for area under the curve (AUC) closely followed by Random Forest and Naïve Bayes. J48 had the least AUC. The corresponding scalar AUC values can be viewed in Table 2

**Figure 2:**
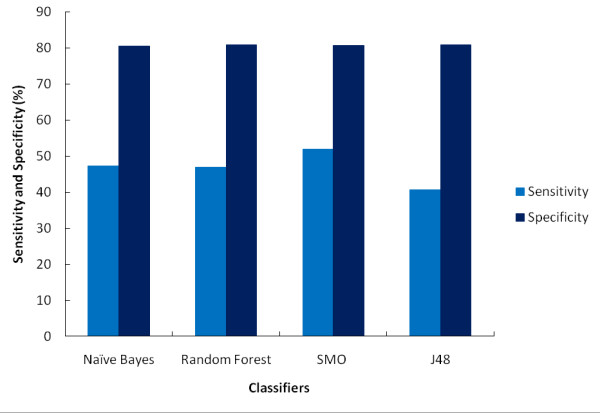
**Sensitivity and Specificity plot of all models**. The Sensitivity and Specificity plot of classifiers revealed an optimal prediction by all models. All the classifiers performed uniformly having high and equal specificity values with SMO being slightly more sensitive than others.

The performance statistics of best classification models obtained with each classifier are represented in Table 2. All the results reported here are based on independent testing and not on the training. Since a number of models were trained on each dataset using different cost settings, best models of each dataset in each classifier category were selected based on various binary classification measurements. All generated models had a controlled FP rate. ROC curve analysis is considered as one of the best and reliable approach for performance characterization of virtual screening protocols therefore, the ROC curve and AUC values are widely employed for evaluating the discriminatory power of virtual screens. The ROC curve analysis from Figure 1 revealed that out of the four classifiers used in this study, SMO covers the maximum area under the curve (i.e. 0.75) followed by Random Forest, Naïve Bayes and J48. An AUC value close to 1 is considered significant in data analytics. In order to make out the classifier's ability to efficiently identify actual positive and negative labels, a measure of Sensitivity (a.k.a. Recall-rate) and Specificity for each dataset was used respectively (Figure 2). An optimal prediction aims to achieve 100% sensitivity and specificity. All classifiers were highly specific in their predictions with specificity more than 80% and in terms of sensitivity SMO appeared to be the most sensitive among all.

**Table 2:** Statistics of best predictive models for AID449762

Classifier*	*TP rate*	*FP rate*	*TN rate*	*FN rate*	*Accuracy*	*ROC area*	*BCR^#^*
**CSC NB**	47.30	19.50	80.50	52.70	80.28%	0.70	63.90
**CSC RF**	47.00	19.20	80.80	53.00	80.58%	0.712	63.90
**CSC SMO**	51.90	19.30	80.70	48.10	80.52%	0.748	66.30
**Metacost J48**	40.60	19.10	80.90	59.40	80.62%	0.61	60.75

*CSC denotes *CostSensitiveClassifier*, ^# ^BCR denotes Balanced Classification Rate

Though all the models built using the four state-of-the-art classifiers had accuracies above 80% but due to the class imbalance problem in the data, BCR was used to assess the robustness of the models. A consistent BCR gave a precise estimation of overall model efficiency as it equally weights the errors within each class. Though all models are observed to have equivalent predictive ability, SMO turns out to be the best among all with high sensitivity, maximum ROC value and highest BCR rate.

The general aim of any virtual screening method is to retrieve a significant portion of true positives from a database being screened over random compound selection. In order to quantify the enrichment produced by *in-silico *screening, we calculated Enrichment Factor (EF) on variable dataset sizes. Popular EFs for early enrichment rates are usually calculated at 1%, 2%, 5% and 10% of the screening database. The EF values obtained with our best model i.e. SMO were 3.7 (EF1%), 4.9 (EF2%), 3.8 (EF5%) and 3.02 (EF10%). These values suggest that our model is able to achieve 3-4 fold enrichment over random screening. Thus for the given dataset under study, SMO is proposed to be the best classifier for identifying inhibitors from axenic culture of *Mycobacterium tuberculosis*.

## Conclusions

In the present analysis of publicly available bio-assay datasets for anti-tubercular activity in vitro, we show that machine learning approaches can be efficiently used to build predictive classifiers for anti-tubercular activities. High AUC values and reasonable BCR rates suggest that these predictive models can serve as an effective filter to screen large chemical libraries. The major caveat of this approach is that the prioritization of the molecules are target-agnostic and may at times would not have any biological correlate given the present understanding of the biological processes and needs to be used in conjunction with other molecular biology techniques to decipher the targets and mechanisms of action. The amenability of a wide variety of bio-assay datasets now in public domain makes it possible to create classifier models based on them. This offers the potential to apply multiple models based on additional properties like toxicity, bio-availability, metabolic processes etc., in conjunction to filter large molecular libraries in-silico before being taken up for biological screens.

## Methods

### Biological assay data

The High Throughput Biological Screen (HTS) data of molecules for anti-tubercular activity (Assay ID: 449762) was obtained from the PubChem data repository maintained by the National Centre for Biotechnology Information (NCBI) [23]. The HTS was based on microdilution Alamar blue assay [24] adapted to 384-well plate format and uses Middlebrook 7H9 broth with glycerol as the growth media. The screen was performed on a compound library which consisted of 3,27,561 compounds. The confirmatory screen (i.e. AID449762) excluded previously known inhibitors from [16,17] and consists of 3,12,901 compounds identified as inactives and only 1937 compounds as actives of which only 117 compounds showed activity < = 1 μM. By the assay definition, compounds that showed > 30% inhibition for at least one concentration in the dose response were defined as "Active". If the inhibition at all doses was < 30% in the Mtb assay, the compound was defined as "Inactive". In the primary screen a compound was deemed "Inactive" if it had a percent inhibition < 70.31%. The chemical structures of both active and inactive compounds were downloaded as SDF files (this information can be found at the following link http://genome.igib.res.in/Mtb_7H9/AID449762.tar.gz).

### Molecular descriptors

Molecular Descriptors were generated for the dataset using the freely available Windows based descriptor calculation software PowerMV [25]. PowerMV provides a software environment for viewing, descriptor generation and hit evaluation and its capacity is only limited by available memory. As the number of compounds (~0.3 Million) in the bioassay used in this study was very large, the entire dataset file was split to smaller SDF files using a perl script available from MayaChemTools [26]. Each of the file was then loaded in PowerMV serially and a set of 179 2D-descriptors corresponding to molecular features were calculated for all the compounds in the dataset AID449762. These descriptors correspond to 147 Pharmacophore fingerprints-bit string descriptors based on bioisosteric principles, 24 Weighted Burden number-continuous descriptors to measure one of the three properties electro negativity, Gasteiger partial charge or atomic lipophilicity, XLogP and 8 Properties-useful for judging the drug-like nature of a molecule like H-bond donors, H-bond acceptors, molecular weight, blood-brain indicator, XLogP etc. The complete list of the descriptors used is provided as Additional file 1: Table S1. The descriptor files were combined into a single CSV (comma separated values) file. Bioactivity values were appended as the last index labeled as 'Outcome' depicting the class attribute which consists of nominal values 'Active' and 'Inactive'.

### Data pre-processing

The merged descriptor file was pre-processed by removing attributes having only one value throughout the dataset *i.e*. bit-string fingerprints containing all 0's or all 1's in them. This was accomplished by applying an un-supervised attribute filter available in the Weka suite of Machine Learning algorithms [27]. Removing non-informative descriptors decreased the dimensionality of the dataset. The dataset was ordered by class. Finally, a bespoke perl script was used to split the data into 80% training cum validation set and 20% test set. The training cum validation set was used to build classification models. A cross-validation (CV) of 5-fold was used during all model building runs. In each iteration of an *n*-fold CV, one fold is used for testing and the other n-1 folds are used for training the classifier. The test results are collected and averaged over all folds. This gives the cross-validated estimation of the resulting accuracy values.

### Machine learning of the dataset

All classification and analyses were performed on the Weka workbench. Weka is a popular open source Java based software that contains implementations of a diverse range of classification and clustering algorithms and a number of other utilities for data exploration and visualization with the flexibility of incorporating new or customized classifiers and components. In this study we present a comparative account of four state-of-the-art classifiers namely Naïve Bayes, Random Forest, J48 and SMO that are trained to build predictive models. A brief description of these algorithms is given below:

#### Random Forest

Random Forests are a combination of tree predictors in which multiple classification trees are constructed from an independent identically distributed random input vector. After a large number of trees are generated, each tree in the forest gives a classification or votes for a class and the most popular class gives the final classification [28]. The main advantage of this method is that it is fast while at the same time, capable of handling of large input variables without over-fitting.

#### Sequential minimal optimization (SMO)

SMO is an implementation of Support Vector Machine (SVM) that globally replaces all missing values and transforms nominal attributes into binary ones. It also normalizes all attributes by default. Unlike the classical SVM algorithm which used numerical Quadratic Programming (QP) as an inner loop, SMO uses an analytic QP step [29]. An SVM is a hyperplane that separates a set of positive examples from a set of negative examples with maximum margin. SMO is conceptually simple, easy to implement and faster in computation. Fitting logistic regression models to the outputs of the SVM could in addition provide probability estimates.

#### J48

J48 implements the decision tree learner algorithm C4.5. It creates a tree data structure that can be used to classify new instances. The leaf nodes contain the class label. Each internal node of the tree contains a test decision result which decides what branch to follow from a particular node. The leaf nodes contain the class label [30].

#### Naive Bayes

This classifier is based on a strong assumption that each descriptor is statistically independent. It learns the conditional probability of each descriptor given the class label. Classification is performed by applying the Bayes rule to compute the probability of a class given particular instance of descriptors and then predicts the class with highest posterior probability [31]. It is one of the most effective and simplest classifier.

### Building classification models

One of the issues with high-throughput biological assays is that the datasets are often skewed on imbalanced. A dataset is termed imbalanced if at least one of the classes is represented by significantly less number of instances than the other. In high-throughput unbiased biological assay datasets, the skew is often towards the inactive set with the actives comprising a minority class. This class imbalance adds to the complexity of the classification problem. Standard error-based classification methods when applied to highly imbalanced data often results in severely skewed predictions that can result in excessively high false negative rate. Therefore, in recent years many strategies have been proposed to derive classification rules for imbalanced data [32]. Introducing misclassification cost on false predictions makes the error-based classifiers cost-sensitive and increases the true predictive ability of the classifier [33]. Setting of misclassification cost is always arbitrary and no generalized rule exists to set the cost.

There are two ways of introducing misclassification cost in classifiers, first to design customized cost sensitive algorithms and second to build a wrapper class that can convert existing base algorithm into cost sensitive one. The later method is commonly referred to as meta-learning [34]. In Weka meta-learning is used to introduce cost sensitivity in base classifiers. *MetaCost *is based on relabeling training instances with minimum expected cost class and then applying the error-based learner to the new training set, generating reliable probability estimates on training examples [35]. This implementation uses all bagging iterations when reclassifying training data and works well for unstable data. *CostSensitiveClassifier *deploys two methods that can be used to introduce cost-sensitivity: reweighting training instances according to the total cost assigned to each class; or predicting the class with minimum expected misclassification cost [36]. In our study we used the former method and thus the *MinimizedExpectedCost *option was set to false.

**Figure 3:**
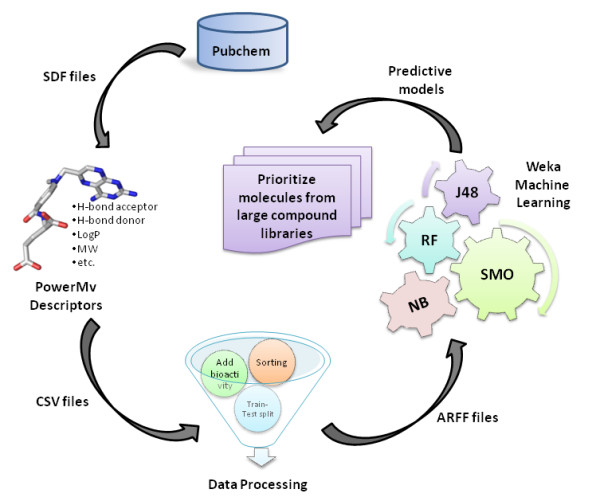
**A Schematic illustration of methodology used**. HTS data was downloaded from PubChem [13] database. Molecular descriptors were calculated with software PowerMv [25]. Resulting data was processed to create train/test files and thereby used generate classification models on the Weka [27] workbench.

The complete methodology to create predictive models that is implemented in the current study is depicted in Figure 3.

### Performance measures

Various performance measures were used to evaluate the results. True Positive Rate (TPR) is ratio of predicted true actives to actual number of actives (i.e. TP/TP + FN), False Positive rate (FPR) is ratio of predicted false actives to actual number of inactives (i.e. FP/FP + TN). Accuracy indicates proximity of measurement of results to the true value. It can be calculated as (TP + TN/TP + TN + FP + FN). Sensitivity (TP/TP + FN) relates to the test's ability to identify positive results whereas Specificity (TN/TN + FP) relates to the test's ability to identify negative results. A test with high sensitivity and specificity has a low error rate. The enrichment factor (EF) represents one of the most prominent performance descriptors in virtual screening. It takes into account the enhancement of the hit rate by a virtual screening protocol compared to a random selection. It can be calculated as the fraction of active compounds found divided by the fraction of screened library. A Balanced Classification Rate (BCR) (0.5*(sensitivity + specificity)) defined as mean of sensitivity and specificity gives a combined criteria of measurement that gives a balanced accuracy for unbalanced datasets. A Receiver Operating Characteristic (ROC) curve is a graphical plot of TPR vs. FPR for a binary classification system. ROC space is defined by FPR and TPR on X and Y axes respectively. The Area under Curve (AUC) value reported by a ROC is equal to the probability that a classifier will rank a randomly chosen positive instance higher than a randomly chosen negative one.

## Authors' contributions

VP under the supervision of VS developed and implemented the work flow. SK and OSDD consortium contributed in building and optimizing the classification models. VP and VS carried out the data analysis and wrote the manuscript. All authors have read and approved the manuscript.

## Supplementary Material

Additional file 1 **Table S1**. Descriptors List. Microsoft DOC file containing a table detailing the list of initial number of total descriptors calculated with PowerMv [25] and the ones removed after data processing and their categorical division. Click here for file
