# Supplementary material for: Computational models for in-vitro anti-tubercular activity of molecules based on high-throughput chemical biology screening datasets
Source: BMC Pharmacol. 2012 Mar 31;12:1. doi: 10.1186/1471-2210-12-1 (PMC3342097; doi:10.1186/1471-2210-12-1)
Supplement: Additional file 1 — Table S1. Descriptors List. Microsoft DOC file containing a table detailing the list of initial number of total descriptors calculated with PowerMv [25] and the ones removed after data processing and their categorical division. [file 1471-2210-12-1-S1.DOC]

**Supplementary Table 1** Division of descriptors calculated for each dataset

| Descriptor category* | Descriptors before data processing | | | Descriptors removed after data processing |
| --- | --- | --- | --- | --- |
| 1.Pharmacophore fingerprints (147) | NEG_01_NEG – NEG_07_NEG  NEG_01_POS – NEG_07_POS  NEG_01_HBD – NEG_07_HBD  NEG_01_HBA – NEG_07_HBA  NEG_01_ARC – NEG_07_ARC  NEG_01_HYP – NEG_07_HYP  POS_01_POS – POS_07_POS  POS_01_HBD – POS_07_HBD  POS_01_HBA – POS_07_HBA  POS_01_ARC – POS_07_ARC  POS_01_HYP – POS_07_HYP  HBD_01_HBD – HBD_07_HBD  HBD_01_HBA – HBD_07_HBA  HBD_01_ARC – HBD_07_ARC  HBD_01_HYP – HBD_07_HYP  HBA_01_HBA – HBA_07_HBA  HBA_01_ARC – HBA_07_ARC  HBA_01_HYP – HBA_07_HYP  HYP_01_HYP – HYP_07_HYP | | | NEG_01_POS  NEG_02_POS  NEG_01_HBA  NEG_02_HBA  NEG_01_ARC  NEG_01_HYP  POS_01_POS  POS_02_POS  POS_01_HBD  POS_01_HBA  POS_02_HBA  POS_01_ARC  POS_01_HYP  HBD_01_HBD  HBD_02_HBD  HBD_01_HBA  HBD_02_HBA  HBD_01_ARC  HBD_01_HYP  HBA_01_HBA  HBA_02_HBA  HBA_01_ARC  HBA_02_ARC  HBA_01_HYP  ARC_01_HYP |
| 2.Weighted Burden  Number (24) | WBN_GC_L_0.25  WBN_GC_H_0.25  WBN_GC_L_0.50  WBN_GC_H_0.50  WBN_GC_L_0.75  WBN_GC_H_0.75  WBN_GC_L_1.00  WBN_GC_H_1.00 | WBN_EN_L_0.25  WBN_EN_H_0.25  WBN_EN_L_0.50  WBN_EN_H_0.50  WBN_EN_L_0.75  WBN_EN_H_0.75  WBN_EN_L_1.00  WBN_EN_H_1.00 | WBN_LP_L_0.25  WBN_LP_H_0.25  WBN_LP_L_0.50  WBN_LP_H_0.50  WBN_LP_L_0.75  WBN_LP_H_0.75  WBN_LP_L_1.00  WBN_LP_H_1.00 | None |
| 3.Properties (8) | XLogP, PSA, NumRot, NumHBA, NumHBD, MW, BBB, BadGroup | | | None |

* Values in brackets depict the number of descriptors calculated in each category
